# Malaria Genomics, Vaccine Development, and Microbiome

**DOI:** 10.3390/pathogens12081061

**Published:** 2023-08-18

**Authors:** Xinzhuan Su, Rachel V. Stadler, Fangzheng Xu, Jian Wu

**Affiliations:** Laboratory of Malaria and Vector Research, National Institute of Allergy and Infectious Diseases, National Institutes of Health, Rockville, MD 20852, USA; rachel.stadler2@nih.gov (R.V.S.); fangzheng.xu@nih.gov (F.X.); jian.wu2@nih.gov (J.W.)

**Keywords:** *Plasmodium*, genetics, genotyping, genome diversity, antigen

## Abstract

Recent advances in malaria genetics and genomics have transformed many aspects of malaria research in areas of molecular evolution, epidemiology, transmission, host–parasite interaction, drug resistance, pathogenicity, and vaccine development. Here, in addition to introducing some background information on malaria parasite biology, parasite genetics/genomics, and genotyping methods, we discuss some applications of genetic and genomic approaches in vaccine development and in studying interactions with microbiota. Genetic and genomic data can be used to search for novel vaccine targets, design an effective vaccine strategy, identify protective antigens in a whole-organism vaccine, and evaluate the efficacy of a vaccine. Microbiota has been shown to influence disease outcomes and vaccine efficacy; studying the effects of microbiota in pathogenicity and immunity may provide information for disease control. Malaria genetics and genomics will continue to contribute greatly to many fields of malaria research.

## 1. A Brief Introduction to Human Malaria Species and Life Cycle

**Figure 1:**
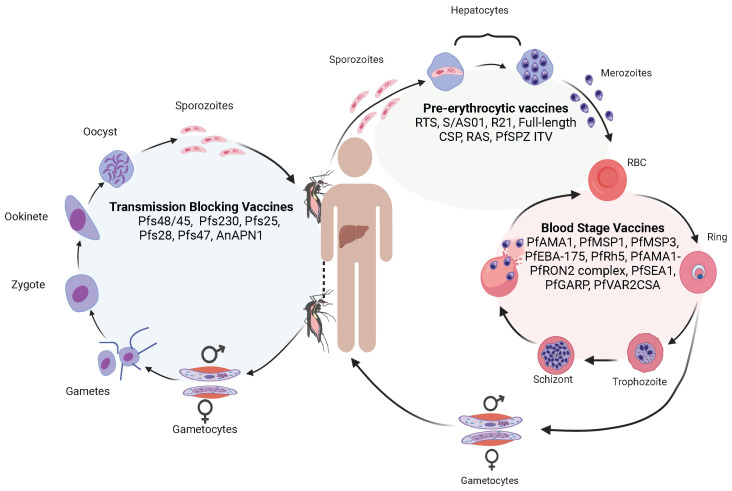
The life cycle of malaria parasites, with major vaccine candidates targeting different developmental stages. The malaria life cycle starts with a mosquito bite injecting sporozoites into the human skin. The sporozoites travel to the bloodstream to invade hepatocytes, where the parasites replicate to produce thousands of merozoites. Some vaccines targeting the liver-stage parasites include RTS,S/AS01, R21, full-length circumsporozoite protein (CSP), radiation-attenuated sporozoites (RAS), and PfSPZ immunization–treatment–vaccination (PfSPZ ITV). Mature merozoites from the liver are then released into the bloodstream again to invade RBCs. Within an RBC, the parasite replicates to produce more merozoites that can invade new RBCs for additional cycles. Some vaccines targeting the *P. falciparum* blood stages include apical membrane antigen 1 (PfAMA1), merozoite surface protein (PfMSP1), PfMSP3, erythrocyte-binding antigen 175 (PfEBA-175), reticulocyte-binding protein homolog 5 (PfRh5), AMA1-rhoptry neck protein 2 (PfAMA1-PfRON2) complex, schizont egress antigen-1 (PfSEA1), glutamic-acid-rich protein (PfGARP), and erythrocyte membrane protein-binding chondroitin sulfate A (PfVAR2CSA). For unknown reasons, some merozoites may develop into male and female gametocytes that differentiate into male and female gametes in the mosquito midgut when another mosquito takes a blood meal. The male and female gametes fertilize to form zygotes that differentiate into motile ookinetes. The ookinetes penetrate the mosquito midgut and develop into oocysts outside the midgut wall. Each oocyst contains thousands of sporozoites that travel to mosquito salivary glands. Transmission-blocking vaccines are aimed at blocking the development of sexual stages within mosquitoes. Vaccines targeting these stages include Pfs48/45, Pfs230, Pfs25, Pfs28, Pfs47, and anopheline alanyl aminopeptidase N (AnAPN1) which is a mosquito midgut protein. When the mosquito bites another vertebrate host, the sporozoites are injected into the new host, starting another cycle.

Human malaria is a disease caused by *Plasmodium* parasites, including *P. falciparum*, *P. vivax*, *P. ovale*, *P. malariae*, and *P. knowlesi*. Whereas *P. vivax* is the most widespread species, *P. falciparum* causes the majority of the mortality, with an estimated 619,000 malaria deaths globally in 2021 [1]. *Plasmodium* parasites generally have a limited host range, e.g., human malaria parasites do not infect rodent hosts and vice versa, although some parasites can infect both humans and non-human primates (for example, *P. knowlesi*). Malaria parasites are single-cell eukaryotic organisms with complex life cycles involving the development of asexual and sexual stages (gametocytes) in vertebrate hosts (humans, non-human primates, rodents, birds, lizards, etc.) and sexual differentiation and fertilization in *Anopheles* mosquitoes [2] (Figure 1). Briefly, when a mosquito takes a blood meal that contains male and female gametocytes, fertilization and genetic exchange can occur in the mosquito midgut. Within the midgut, male and female gametocytes (1n) differentiate into male and female gametes that fertilize to produce zygotes (2n), which then turn into motile ookinetes (4n) after DNA replication. The ookinetes penetrate the mosquito midgut wall to develop into oocysts containing thousands of sporozoites (1n) after meiosis. Mature sporozoites move to the mosquito’s salivary glands and are injected into the skin when the mosquito bites a human host. The motile sporozoites travel from the skin to the liver, where they develop into schizonts containing thousands of merozoites. Mature schizonts release the merozoites into the bloodstream to invade erythrocytes. Within the erythrocytes, parasites develop into rings, trophozoites, and finally merozoites that can infect new erythrocytes when released. Some merozoites will convert into sexual stages, producing male and female gametocytes. Fertilization, DNA replication, and genetic recombination provide biological platforms for studying parasite population structure, transmission dynamics, genetic exchange, and evolution using genomic and genetic tools. The multiple developmental stages in the complex mosquito life cycles also allows for the identification of various potential targets for vaccine development (Figure 1).

## 2. Malaria Genetics and Genomics

**Figure 2:**
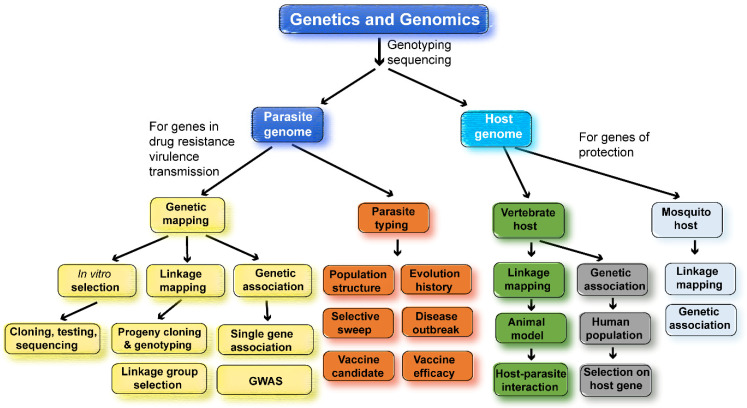
Summary of major approaches and applications in genetic and genomic studies of malaria parasites. Many genetic and genomic approaches have been employed to study the malaria parasite populations, transmission, molecular evolution, drug resistances, host–parasite interaction, and vaccine targets. Genetic crosses can be performed using different strains of malaria parasites to map parasite genes conferring drug resistance or encoding vaccine targets. Similarly, genetic crosses of inbred mice will allow the mapping of host-protective genes. DNA collected from patient blood samples can be used in association studies, either using a single candidate gene or genome-wide association study (GWAS), to identify parasite and host genes of interest and/or to study parasite population structure, origin, molecular evolution, and drug-selective sweeps. Using animal models, it is also possible to study host–parasite genetic interactions, linking host gene response/expression to parasite genes using trans-species expression quantitative locus analysis (ts-eQTL) [4]. When identifying drug-resistant genes, parasites can be selected under drug pressure in vitro (*P. falciparum*) or in vivo (rodent *Plasmodium* species), and genome sequences from the parasites before and after selection can be compared to identify mutations selected using drug treatment. Similar genetic approaches have also been employed to map mosquito genes conferring resistance to malaria infections.

Genomics is defined as “the study of the functions and interactions of all the genes in the genome” [3]. In infectious diseases, genomics includes interactions between the pathogen genomes and their hosts. Large amounts of genomic data are now available, such as genome sequences, proteomics, transcriptomics, and metabonomics from many species or strains of malaria parasites and their hosts. These data are being used to study gene function, gene–gene interaction, genotype–phenotype association, and genetic testing. Epidemiological principles and genomic methods can also be applied to discover genes, to characterize parasite populations, to track parasite origin and disease outbreak, to study parasite evolution history, and to improve vaccine efficacy. The major approaches and applications in malaria genetics and genomics research are summarized in Figure 2.

### 2.1. Genetic Typing and DNA Sequencing Methods Used in Malaria Genetic and Genomics

Based on the types of genetic variations, various genotyping methods have been developed to study malaria parasite populations, transmission, evolution, and gene function through linkage and association mapping. Electrophoretic variants of enzymes were first introduced to study polymorphisms in rodent malaria parasites [5]. Pulsed-field gel electrophoresis (PFGE) was then employed to show polymorphisms of tens to hundreds of kilobases in size in different *P. falciparum* isolates [6,7]. Based on restriction enzyme digestion, agarose gel electrophoresis, blotting, and the hybridization of radio-labeled probes, restriction fragment-length polymorphism (RFLP) analysis was used to study recombination rates and linkage groups in a genetic cross [8]. Later, amplified fragment-length polymorphism (AFLP) analysis was developed to genotype and map genes in *Plasmodium c. chabaudi* parasites [9,10,11]. The AFLP technique also involves digestion of parasite DNA, followed by the ligation of oligonucleotide adapters to the digested DNA, the amplification of the digested DNA fragments, the gel separation of the amplified fragments, and the visualization of the amplified products [9]. Based on the abundance of microsatellites (or simple sequence repeats) in the AT-rich *P. falciparum* genome, PCR amplification of DNA with a small-size polymorphism (microsatellite) was established to determine the type of *P. falciparum* isolates [12,13] and to map a determinant linked to chloroquine resistance [14]. With the advances in DNA sequencing technologies, large numbers of single-nucleotide polymorphisms (SNPs) were identified using DNA sequencing [15,16,17]. Based on the SNPs identified from sequencing, microarrays were developed to genotype *P. falciparum* and *P. yoelii* parasites [18,19,20,21]. With the reduced costs of DNA sequencing, genome sequencing of parasite DNA samples from patient blood is becoming a practical and popular method of performing genetic studies of malaria parasites [22,23].

DNA sequencing has been categorized into generations. DNA sequencing began using low levels of radioactive isotopes in DNA polymerization reactions [24,25]. The incorporation of radioactive ddNTP nucleotides into the DNA prevented further DNA extension. DNA fragments were visualized using electrophoresis gels. The sequencing reads were generally small—less than one kilobase (kb). Because of the AT-rich *P. falciparum* genome, sequencing using the isotope methods was very challenging initially. The second-generation sequencing used a luminescent method that measured pyrophosphate synthesis during amplification. This decreased the cost per base sequenced and allowed for higher throughput [26,27]. Light is released when a nucleotide is incorporated, and signals of four different colors representing four nucleotides are recorded. This method allowed for millions of fragments to be sequenced simultaneously per run. However, due to the amount of light produced per reaction, DNA fragments have to be amplified before sequencing reactions. The third-generation sequence allowed for the sequencing of single molecules and therefore eliminated the need for DNA amplification before sequencing [28]. The third-generation sequencing increased the read lengths and decreased the cost and time further. The rise of technologies has enabled the sequencing of the genomes of large numbers of malaria parasites and their hosts. The genotyping and sequencing methods are the key factors that have driven research in malaria genomics.

### 2.2. Genomes and Chromosomes of Plasmodium Parasites

The genomes of many *Plasmodium* species infecting humans, nonhuman primates, rodents, and birds have been sequenced. The first *Plasmodium* genome to be sequenced was the genome of the 3D7 clone of *P. falciparum* in 2002 [29]. The sequencing revealed an AT-rich genome of 22.8 Mb containing 14 chromosomes and 5300 predicted genes. It is now known that all species of malaria parasites have 14 chromosomes consisting of ~20–35 Mb of DNA, a linear 6 kb mitochondrial genome, and a 35 kb circular plastid genome [30,31,32,33,34,35,36,37,38,39,40,41]. There are also extensive gene syntenies among the malaria parasite species [42,43,44]. Similar genomic structure is an important common feature of the *Plasmodium* parasites. In contrast, the genome sequences of *Plasmodium* parasites can be very different in GC content, genome size, and the number of multigene families. For example, the *P. falciparum* genome is highly AT-rich (~80% AT), whereas the *P. vivax* genome is relatively more GC-balanced (~58% AT) [30,45], which suggests that *Plasmodium* parasites have a long and complex evolutionary history [40,46]. The genomes of many parasite strains or isolates of various *Plasmodium* species, particularly for *P. falciparum* and *P. vivax,* have been sequenced, revealing diverse genomes with large numbers of SNPs and indels within a single parasite species [40,47,48]. Like many other microorganisms, the chromosomes of the parasites can be divided into highly variable subtelomeric regions and conserved central regions [40,49]. The subtelomeric regions are known to include hypervariable repetitive sequences that often cause errors in genotyping and sequence alignment. It is not reliable to use the SNPs from the subtelomeric sequences to infer parasite ancestry or population structure due to frequent recombination.

### 2.3. Within Species Genetic Polymorphisms

Genetic polymorphisms within individual malaria parasite species include chromosome-size polymorphisms, variations in gene copy number, SNPs, indels (insertion or deletion of nucleotides), and nucleotide modifications. Chromosome-size polymorphisms were recognized in the 1980s after the separation of parasite chromosomes in PFGE [5,50,51]. Chromosome-size polymorphisms largely occur due to the deletion and translocation of chromosomal end segments [51]. Variations in the copy number of gene families also contribute to genetic differences in the parasite strains [44,52,53], and these gene families likely play a major role in parasite virulence and immune evasion [54,55,56,57,58,59]. Large numbers of SNPs and indels have been identified from many *Plasmodium* species, such as *P. falciparum*, *P. vivax*, and rodent malaria parasites, through the genome sequencing of parasite isolates or strains [18,40,48,60]. Recently, more than 4.5 million variable positions, including over 3 million SNPs and indels, were identified from 1895 *P. vivax* isolates collected from 88 worldwide locations [48]. For *P. falciparum*, a total of 3,125,721 SNPs and 2,742,938 indels were reported from genome sequences of ~20,000 parasite isolates after excluding variants in subtelomeric and internal hypervariable regions, as well as the mitochondrial and apicoplast genomes [47,61]. For an estimated 23 Mb *P. falciparum* genome, there is approximately one polymorphic site per four bp sequences. However, the polymorphisms are not evenly distributed throughout the genome. Additionally, most of the polymorphic minor alleles are low-frequency (<5%), occurring in one or several parasites and thus suggesting recent mutations. Genes encoding immune and drug targets are likely to have more SNPs due to immune and drug selections [60,62,63], and microsatellites are often found in AT-rich repeat regions [11,12]. Copy number variation can occur under drug pressures; the gene-encoding *P. falciparum* multidrug resistance 1 (PfMDR-1) can increase its copy number under mefloquine pressure [64,65]. Compared with SNPs, microsatellites generally have higher mutation rates and multiple alleles (in contrast to two alleles for most of the SNPs) and can be used to study recent evolutionary events in parasite populations. Because of maternal inheritance and limited recombination, SNPs and indels from the mitochondrial and plastid genomes are better markers for tracking parasite origin and studying parasite evolution [66,67,68,69,70]. Many genetic polymorphisms can influence gene functions and parasite development. On the other hand, they also provide tools for studying parasite evolution, population dynamics, gene function, and host–parasite interactions.

### 2.4. Identification of Drug Resistance Genes and Monitoring the Origin and Spread of Drug Resistance

Genetic and genomic tools have been employed to study malaria drug resistance extensively. The studies can be classified into three major directions: first, the identification of drug-resistant genes using linkage and association mappings, including genome-wide association study (GWAS); second, tracking and monitoring the origins and spread of drug-resistant parasites (drug-selective sweeps) using samples collected from patients; and third, drug selection in vitro or in vivo and genome sequencing or mapping of new mutations under drug pressure (Figure 2). These fields have been the most active in malaria research and will continue to receive great attention in the future. There have been many reviews on malaria drug resistance, the genetic mechanisms of resistance, and genetic/genomic approaches used to study drug resistance [71,72,73,74,75,76,77,78]. Readers who are interested in these subjects can consult the reviews listed above.

### 2.5. Host Genetic Polymorphisms and Protection against Malaria

Genetic variation in parasites leads to different disease outcomes, but host genetics can also impact the severity of the infection. For example, mutations in hemoglobin—leading to sickle cell amenia—or the absence of the Duffy antigen receptor give the host better survival outcomes. Sickle cell amenia is caused by a single amino acid mutation in hemoglobin, which leads to red blood cells having long crescent shapes [79,80]. Sickle red blood cells increase host survival via multiple means, including the increased clearance of infected cells by the spleen [81,82] and the trafficking of parasite proteins to the erythrocyte membrane [83]. While the sickle cell anemia trait led to the clearance of infected cells by the host, the absence of the Duffy antigen receptor prevented erythrocyte infection by *P. vivax* [84,85,86]. Infections by *Plasmodium* parasites placed selective pressure on the human host, resulting in mutations in the human genome.

## 3. Malaria Genomics and Vaccine Development

**Figure 3:**
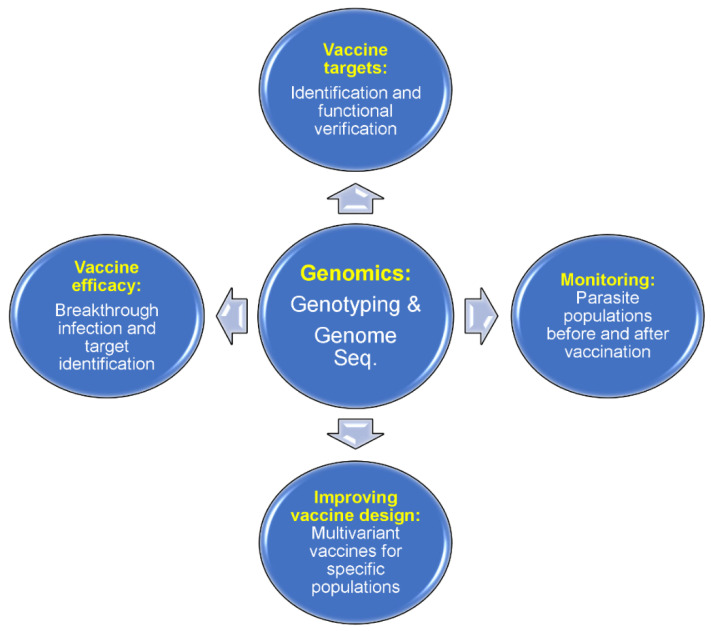
Applications of malaria genomics in vaccine development. Genome sequences from parasite isolates can be studied for potential protective antigens. A candidate antigen can be tested for the efficacy of its protection in animal models before trials on humans. An antigen sequence in parasite populations from a specific endemic region can be surveyed for diversity, and a multivariant vaccine (with reduced diversity in a local setting) can be designed based on major alleles present in a specific location such as South American countries. Vaccine efficacy can be evaluated by surveying allele frequencies before and after vaccination. For whole-organism vaccines, a survey of genome-wide polymorphisms before and after vaccination (or vaccine vs placebo recipients) may help to identify the protective vaccine targets. Finally, there is a chance that a partially effective vaccine may change the parasite populations circulating in an endemic region, which may alter the virulence of parasite populations. New mutations may occur after vaccination, reducing the effectiveness of a vaccine.

There are many malaria vaccines under various stages of development. Malaria vaccines can be divided into three major categories on the parasite developmental stages, including pre-erythrocytic vaccines (anti-liver stages), blood-stage vaccines, and transmission-blocking vaccines (anti-sexual stages) (Figure 1). Among the vaccines against each stage, there are single-molecule or multiantigen vaccines, multistage vaccines, and whole-organism-based vaccines [87,88,89,90,91,92,93,94,95]. The most successful malaria vaccine against *P. falciparum* parasites developed so far is the RTS,S/AS01 which consists of 18 copies of the central repeat and the C-terminal domain of PfCSP fused to the hepatitis B virus surface antigen (HBsAg) [93,94]. The RTS,S vaccine has been recommended for human use by the WHO [95]. However, a sterile malaria vaccine (or 100% protection) is not available currently; more research and development are required to achieve the goal of eradication of malaria through vaccination. Genetic and genomic tools have the potential to contribute significantly to the development of an effective malaria vaccine, providing information for the identification of vaccine targets, improving vaccine design, evaluating vaccine efficacy, and monitoring potential vaccine “resistance” or escape (Figure 3).

### 3.1. Identification of Vaccine Candidates

Genomic information and tools can be employed to identify candidate vaccine targets and predict T/B cell epitopes [96,97]. Computational methods can be used to search parasite genomes for putative surface-exposed or secreted proteins in an approach called “reverse vaccinology” [96]. A multivalent vaccine was developed against meningitis and sepsis caused by the bacteria *Neisseria meningitidis* using this approach [98]. Recombinant proteins from candidate genes identified through genome searches can be expressed, purified, and used to immunize mice to produce antibodies. Selected candidates can be checked for sequence conservation across a panel of different parasite strains to identify conserved epitopes or relatively conserved antigens. The ability of the antibodies to protect against a microorganism or parasite are tested in functional assays such as growth inhibition or transmission-blocking assays [99,100] and eventually evaluated in animal models and humans.

Most of the malaria antigen genes are highly polymorphic due to balancing selection, and searches for hotspots of genome diversity may lead to potential unknown immune targets. A survey of 3539 *P. falciparum* genes for polymorphisms identified some highly polymorphic loci and candidate genes. This included some genes of unknown function that were confirmed to encode antigens recognized by the human immune sera [60]. In another study, a genomic survey of 65 *P. falciparum* isolates from West Africa identified 2853 genes that contained three or more SNPs [63]. Genes with strong evidence of balancing selection and with peak expression at the stage of red blood cell (RBC) invasion were highlighted, including members of *clag*, *PfMC-2TM*, *surfin*, and *msp3-like* gene families. The genes under balancing selection can be prioritized for functional study and testing for use in potential vaccines. Unfortunately, highly polymorphic proteins may not be ideal vaccine candidates because of parasite-strain-specific immunity and immune evasion of parasites with variant alleles. However, it may be possible to search for protective antigens that are relatively conserved in parasite populations across geographic regions. Indeed, antigen candidates that had low naturally occurring sequence variation and high coverage across diverse bacterial populations were found after sequencing 2083 group A *Streptococcus pyogenes* bacterial isolates [101]. The most successful molecular malaria vaccine developed so far, RTS,S, is based on a highly-conserved tandem repeat tetrapeptide (NANP) in the central repeat region of the CSP and a C-terminal region that contains T- and B-cell epitopes, although the central repeat region itself is highly variable in size among parasite isolates [102]. The use of conserved tandem repeat sequences in a variable region in size as a vaccine may be good practice for malaria parasites because there are many proteins with repeated motifs in the parasite genomes, particularly in the *P. falciparum* genome. With genome sequences from over 20,000 *P. falciparum* isolates available, it is now possible to search for candidate vaccine targets that have relatively conserved motifs in most of the strains and are expressed in important parasite stages such as merozoite or sporozoite [47,61]. 

In addition to diversity-based search, experimental genetic crosses of parasites and genome-wide analyses have also been used to identify genes that encode targets of host immunity. In one study using progenies of *Plasmodium chabaudi chabaudi* genetic crosses, Cheeseman et al. developed a genetic approach called linkage group selection to identify target antigens of strain-specific protective immunity (SSPI) against malaria [103]. The analysis of the frequencies of genome-wide polymorphisms from 35 SSPI selection events on different populations of progeny pools identified a 79 kb genomic region on chromosome 8 as the region controlling SSPI. A gene encoding the merozoite surface protein 1 (MSP-1) within the locus was found to account for >60% of genetic polymorphism and was most frequently under the greatest selection by SSPI [103]. The results are consistent with the fact that MSP-1 is a known malaria antigen that is being used to develop a malaria vaccine [104]. In another study, a key molecule expressed on the *P. falciparum* merozoite, known as PfRh5, was identified through genetic mapping by the ability of the parasite to invade *Aotus* monkey erythrocytes as a phenotype [105]. PfRH5 plays an important role in erythrocyte invasion and has become a promising next-generation blood-stage vaccine candidate [106,107].

In addition to candidate antigens, large numbers of linear and discontinuous epitopes of T and B cells and MHCII have been detected in the genomes of *Plasmodium* species using genomic tools [108]. Many experimentally validated T and B cell and MHCII epitopes were found in well-known antigens such as RIFIN (repetitive interspersed families of polypeptides), STEVOR (subtelomeric variable open reading frame), PfEMP1 (*P. falciparum* erythrocyte membrane protein 1), MSPs, EBA (erythrocyte-binding antigen), and CSP proteins [108]. Additionally, approximately 50% of the epitopes from each species were predicted to induce IFN-γ, and approximately 26% of unique epitopes were predicted to have IL-10-inducing potential. The epitope analyses of vaccine candidates provide information that is valuable when designing a better vaccine.

### 3.2. Improving Vaccine Design

A difficulty in developing an effective malaria vaccine is the high level of polymorphism in candidate antigens, which may result in immune escape due to strain-specific immunity. Genomic information can be used to inform the design of malaria vaccines. A molecular vaccine can include multiple alleles of a target antigen to generate antibodies against different parasite strains circulating in a local endemic region as this will allow it overcome immune escape by parasite variants. For example, limited allelic variants (mostly three alleles) were found in genes encoding CSP, MSP-1, AMA-1, LAS-1 (liver stage antigen 1), and TRAP (thrombospondin-related anonymous protein) in 139 *P. falciparum* isolates collected from Amazon basin in Loreto, Peru [109]. A multivariant vaccine consisting of three major alleles for each specific antigen may be sufficient to cover most of the parasite variants in the regions. The *P. falciparum* populations in South America are relatively homogeneous due to drug-selective sweeps [110,111]. Parasite populations in Africa are generally more diverse, and more antigen variants are necessary to counter the diverse populations in Africa. However, for some antigens, genetic variation is relatively limited. A recent study showed a low level of genetic polymorphisms in the PfRH5 antigen in Lagos, Nigeria [112]. Sequence analysis revealed three haplotypes of PfRH5 with a negative Tajima’s D of − 1.717 and a dN/dS value of 0.011 (±0.020), suggesting a population expansion after a recent bottleneck and/or the selection on this gene. A vaccine with sequences covering the three PfRH5 haplotypes can be designed to vaccinate the human populations in the Lagos region. A region-specific vaccine based on the specific target antigen variants can be developed to protect the local human populations after a survey of parasite populations. This approach will be more practical for parasites in South America due to relatively homogenous parasite populations.

### 3.3. Evaluation of Vaccine Efficacy or Identification of Unknown Vaccine Targets

In vaccine trials, it is often necessary to know whether the parasites from patients who received a vaccine have the same genotype as the ones used in vaccine development. For a molecular vaccine based on a single-parasite antigen, it is relatively easy to sequence the antigen coding gene in parasite isolates collected from vaccinated individuals. Several studies investigated the RTS,S vaccine efficacy through sequencing or genotyping the vaccine targets from vaccine recipients and compared the frequencies of matched (3D7 strain) or mismatched sequences [113,114,115,116]. In particular, analysis of polymorphic sites and haplotypes within the CSP gene in samples from an RTS,S/AS01 vaccine trial found that the 1-year cumulative vaccine efficacy was 50.3% against clinical malaria with parasites matching the vaccine sequence in the entire CSP C-terminal, as compared with an efficacy of 33.4% against those with mismatching sequences [116].

For a whole-parasite vaccine such as attenuated sporozoite vaccines [90,117], determining the type of parasite genome using multiple genetic markers or even performing whole-genome sequencing may be necessary before a vaccine trial. In a recent study, whole-genome sequencing was performed to generate de novo genome assemblies for the vaccine strain (NF54) and other heterologous strains used in controlled human malaria infection (CHMI) trials in order to investigate genotypic and immunologic differences [118]. Based on the long genetic distance between the vaccine strain PfNF54 of West Africa and a Brazilian isolate Pf7G8 and drawing from the evaluation of host responses to vaccine trials, it was proposed that Pf7G8 could act as a stringent surrogate for *P. falciparum* parasites in Africa [119].

Comparison of the genome sequences of the vaccine strain and the strains before and after vaccination may help to identify some key protective epitopes or antigens in whole-organism vaccines such as *P. falciparum* sporozoite (PfSPZ) vaccines. For a whole-organism vaccine, the protective epitopes or antigens are generally unknown. An approach called ‘sieve analysis’ has been used to compare viral or parasite genetic sequences between vaccine and placebo recipients in order to study breakthrough populations, vaccine efficacy, and potentially identifying vaccine targets [116,120,121]. In the analysis, genetic and statistical approaches are used to measure the dissimilarity between the vaccine sequence and sequences isolated from trial participants, and the dissimilarities are then compared between vaccine and placebo recipients. The radiation-attenuated PfSPZ vaccine has been shown to protect > 90% of subjects against homologous CHMI, although it offers lower rates of protection against heterologous CHMI [122,123,124,125]. However, the critical protective targets or epitopes for homologous protection are currently unknown. Sequencing the genomes of parasite isolates before and after PfSPZ vaccination (if available) and/or between vaccine and placebo recipients may allow for the identification of a collection of protective epitopes via a concept similar to the linkage group selection method used in genetic crosses [126].

### 3.4. Monitoring Potential Vaccine Selection and Escape

Breakthrough infections in fully vaccinated individuals have been reported for vaccines against SARS-CoV-2 due to ineffective immune responses, waning immunity, or escape from immune recognition by viral evolution [127]. Malaria vaccines such as RTS,S do not induce sterile immunity, and infections of vaccinated individuals are likely to occur. Genome sequencing or genotyping using multiple polymorphic genetic markers can be employed to distinguish vaccine breakthrough parasites from new infections of parasites with similar genetic backgrounds, particularly for those patients vaccinated with a whole-organism vaccine. Partial protective vaccines like RTS,S put pressure on parasite populations and select parasites with CSP alleles that are different from those used in vaccine development. Genomics tools also help to monitor the potential emergence of “vaccine-escaping” parasite strains, which may become a challenge in malaria control. There are no solid data showing that the depletion of a parasite strain with a specific antigen allele will change parasite virulence or transmission capability. However, studies of rodent malaria species or strains clearly show that various degrees of virulence and disease severity (phenotype) can be caused by different strains of parasite species [128,129]. In human infections, disease severity is often the result of infections with a mixture of parasite strains. It is likely that strains of *P. falciparum* or *P. vivax* circulating in a human population have different levels of virulence and cause different disease pathologies. For example, the carriage of the msp1-MAD20 or msp2-FC27 allele family was associated with increased susceptibility to severe malaria [130]. Vaccine-targeted depletion of a parasite population or an allele in an individual may change the dynamics of parasite population structure and disease outcomes, particularly when a virulent gene is physically linked to the vaccine target gene, or when the vaccine antigen is a virulent factor itself. Indeed, an antigen used in a vaccine is the target of host immune responses, and different alleles of the antigen may stimulate different host responses. This effect of vaccine pressure may lead to decreased vaccine effectiveness. By surveying changes in the parasite’s genome over time, we can monitor potential parasite population dynamics in the vaccinated regions and predict the emergence of new parasite populations and possibly vaccine efficacy.

### 3.5. Screening for B Cells Producing Protective Monoclonal Antibodies

Monoclonal antibodies (mAbs) produced by a single B cell population can be used as prophylactic or therapeutic agents against infecting microbes, and exciting results have been reported when using monoclonal antibodies against malaria [131,132,133,134,135,136]. The identification and cloning of monoclonal antibodies that are protective against malaria relied on protocols that allow the efficient amplification, sequencing, and expression of many single B cells from malaria patients [137]. Again, tools of efficient DNA sequencing and genetic analyses are critical to identifying which clonal cells produce effective antibodies for prophylaxis and treatment of malaria.

## 4. Malaria, Microbiome, and Vaccine Efficacy

An emerging field taking advantage of high-throughput genomic technologies and biocomputational analyses is studying the association of and interaction between microbiomes and various diseases. Microbial communities that inhabit different body parts such as the intestine and skin have been studied intensively for their association with various diseases in recent years [138,139,140], including malaria [141,142,143]. The estimated number of microbial genes associated with the human body is estimated to be 2–20 million, or approximately 100X the ∼20,000 human genes, and the development of high-throughput genome sequencing technologies and computational methods makes it possible to analyze the genomic data from microbiota [144].

**Figure 4:**
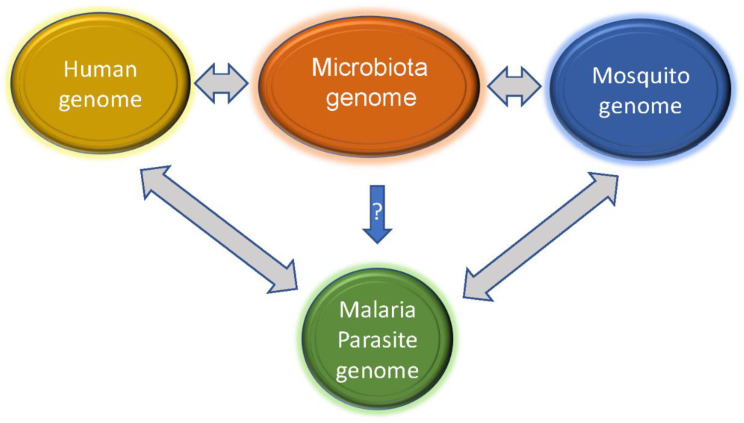
Interactions of microbiota genomes with those of human and mosquito can influence the host responses to malaria parasite infections. Metabolites and components such as nucleic acids released from the microbes may trigger and/or alter the host (human and mosquito) immune responses, although whether there are microbes that can directly inhibit or enhance malaria parasite development is not clear. On the other hand, malaria parasite infections may also change host immunity and microbial populations in the hosts, allowing some commensal populations to become pathogenic.

Although malaria parasite transmission typically requires and interacts with a vertebrate host and a mosquito vector, microbiota within the vertebrate host (human, mouse, or others) and mosquito can also influence host metabolism and immunity, thereby altering the outcomes of parasite growth and transmission (Figure 4). Elucidating host microbiota and their dynamics during malaria parasite infections may reveal molecular mechanisms that can be explored to develop better disease treatment and management. Studies of both rodent malaria models and human malaria have shown that gut microbiota composition is a factor in disease progression [141,142,143]. For example, the same strain of mice from different vendors infected with the same parasite had profound differences in disease severity, and differences in gut microbiota contributed to the differences in disease susceptibility [145]. Further, germ-free mice transplanted with cecal contents from ‘resistant’ or ‘susceptible’ mice had low and high parasite burdens, respectively. The ‘resistant’ mice had increased abundances of *Lactobacillus* and *Bifidobacterium* microbes and an elevated humoral immune response compared with susceptible mice. Additionally, the use of antibiotic treatment and fecal microbiota transplant to change the gut microbiota in outbred Swiss Webster mice also altered the susceptibility to *P.* c. *chabaudi* infection and pregnancy outcomes [146]. On the other hand, infection by *Plasmodium* parasites can induce alterations in the composition of the gut microbiota [143]. *Plasmodium berghei* (PbA) infection decreased the numbers of *Bacteroidetes* and *Verrucomicrobia* species in the mouse gut but increased the numbers of *Firmicutes* and *Proteobacteria* species [147].

Increasing evidence from clinical studies and animal models has also shown that the composition of the gut and skin microbiota plays a critical role in modulating immune responses, disease severity, and vaccine efficacy [148,149,150]. The composition of stool bacteria at the beginning of malaria transmission season was associated with susceptibility to *P. falciparum* infection [151]. Similarly, stool bacteria populations correlate with the severity of malaria in Ugandan children [152]. Children with malaria often have changes in the immune cell architecture of the spleen, which may affect the immunological function of the spleen against encapsulated bacteria [153]. *Plasmodium* infection can also lead to gut microbiota-dependent increases in splenic germinal center (GC)-associated immune cells in mice and in the titer of parasite-specific antibodies [154]. Malaria parasite sequestration in the lungs of PbA-infected mice results in sustained immune activation and production of the anti-inflammatory cytokine IL-10 by T cells, compromising microbial control and leading to severe lung disease [155]. Interestingly, modulation of mosquito microbiota by the anti-microbiota vaccination of birds against commensal *Enterobacteriaceae* disrupts *P. relictum* development within midguts and salivary glands of the vector *Culex quinquefasciatus* [156]. An increased ratio of *Enterobacteriaceae* to *Bacteroides* species was also observed in the responders of a vaccine against rotavirus in a study in Ghana [157]. The differences in the compositions of bacteria nucleic acid and/or lipopolysaccharides may play a role in immune responses during infection or after vaccination. Monitoring the dynamics of the host microbiota on the skin and in the gut using genomic tools may be necessary in order to better understand and improve vaccine efficacy.

Interestingly, the microbiota of the mosquito vector can also affect the transmission of the malaria parasite. One study found that the midguts of *An. stephensi* had *Proteobacteria* as the most dominant population among the three laboratory-reared strains of *An. Stephensi*, and that antibiotic treatment increased susceptibility to PbA infection [158]. Similarly, antibiotics in ingested blood enhanced the susceptibility of *Anopheles gambiae* mosquitoes to malaria infection by disturbing their gut microbiota [159]. In a follow-up study, the vectorial capacity of malaria mosquitoes was found to be dependent on antibiotics in the ingested blood [160]. Azithromycin could decrease *P. falciparum* infection load and mosquito lifespan, whereas doxycycline increased the parasite load at high concentrations. Additionally, gut bacteria may impair the effectiveness of mosquito control approaches using insecticide bacteria such as *Bacillus thuringiensis* subspecies *israelensis* (Bti) or *Wolbachia* to alter the fitness or inhibit insect reproductive organs of infected insects [161]. Therefore, studies of malaria genomics need to include the interactions of malaria parasites, their hosts, and the microbiome. There is increasing interest in studying the interaction of malaria and microbiota, and the continuous advance of genomic technologies and computational power will certainly advance the field of malaria and microbiota studies.

## 5. Conclusions and Perspectives

Malaria genetics and genomics are important approaches for studying disease mechanisms, host–parasite interaction, gene function, drug resistance, parasite population structure and transmission, and for developing better vaccines. Although great progress has been made in malaria genomics and the applications of genomics in studying various aspects of the disease, there are some areas where exciting discoveries may be obtained by applying genomic methods. In particular, analysis of parasite populations before and after vaccination (vaccine and placebo recipients) using a whole-organism vaccine may lead to the detection of immune targets if the vaccinated individuals are infected with multiple parasite strains. Multiple infections with two to eight *P. falciparum* clones were frequently detected in Africa [162], and diverse circulating parasite strains with frequent occurrence of multiple infections were observed in Nanoro, Burkina Faso [130]. If a vaccine removes some specific parasite strains, potential selection valleys can be detected. The presence of polymorphisms in vaccine molecules is another problem in developing an effective vaccine. It may be possible to design a vaccine that targets the local parasite populations after genotyping parasites in an endemic area. This approach may be practical for countries in South America where parasite populations are less diverse than those in Africa due to selective sweeps by chloroquine and other drugs [110,111]. After the selection of candidate antigens, parasite population surveys can be conducted to identify major alleles circulating in the local populations. A multivariant vaccine with limited variant sequences can be designed based on the major alleles in the endemic region. Finally, the genomes of microbiota have been shown to play a critical role in shaping malaria disease severity and vaccine efficacy, which brings a third dimension to the study of malaria genomics (in addition to parasite and host genomes). The study of microbiome genomics and interactions with malaria parasites and their hosts will enhance our understanding of malaria pathogenesis and the mechanism of disease severity. It can be expected that genomic and genetic tools will continue to contribute greatly to vaccine development and malaria elimination.

## Data Availability

All data are included in the article.
